# The Toxicological Assessment of Content and Exposure of Heavy Metals (Pb and Cd) in Traditional Herbal Medicinal Products with Marshmallow Root (*Althaea officinalis* L., radix) from Polish Pharmacies

**DOI:** 10.3390/toxics10040188

**Published:** 2022-04-13

**Authors:** Kamil Jurowski, Mirosław Krośniak

**Affiliations:** 1 Institute of Medical Studies, Medical College, Rzeszów University, Al. mjr. W. Kopisto 2a, 35-959 Rzeszów, Poland; 2 Department of Food Chemistry and Nutrition, Medical College, Jagiellonian University, Medyczna 9, 30-688 Kraków, Poland; miroslaw.krosniak@uj.edu.pl

**Keywords:** EI—elemental impurities, THMP—traditional herbal medicinal product, ICH Q3D—International Council for Harmonization of Technical Requirements for Pharmaceuticals for Human Use, PDE—permitted daily exposure, PTE—potentially toxic elements

## Abstract

The level of potentially toxic elements (PTE) in phytopharmaceuticals can be a potential threat to human health through the food chain. The traditional herbal medicinal products (THMPs) with the marshmallow root (*Althaea officinalis* L., radix), which we can find in European pharmacies, are widely used among the European population. However, recently, voices have been heard in the public about alleged PTE occurrence. In this article, the levels of Pb and Cd impurities were measured in samples of THMPs with marshmallow root available in Polish pharmacies (*n* = 10). Our proposed toxicological approach was based on two important issues. The first was PTE levels (Pb: 1.60–6.80 μg/L and Cd: 0.80–3.81 μg/L presented as raw results) in comparison with the permissible limit set by FAO/WHO for medicinal herbs (10,000 µg/kg for Pb and 300 µg/kg for Cd) and also ICH Q3D guidelines (0.5 μg/g for Pb and also Cd). The second was the estimation of exposure of investigated PTE in a single dose and daily dose for each THMP. It should be noted that the content of analyzed heavy metals in a single dose of analyzed products was very low, and hence is not a threat for patients. The obtained daily intake of heavy metals impurities in comparison with PDE values confirms the safety of all analyzed phytopharmaceuticals (all of the samples meet the standards of the ICH Q3D guideline). It can be summarized that each of the analyzed THMP with marshmallow root available in Poland are safe for the patients. Based on literature review, this article is the first study about heavy metals impurities level in final THMPs with *A. officinalis* L., radix (marshmallow root) available in European pharmacies.

## 1. Introduction

Lead and cadmium are potentially toxic elements (PTE), but usually scientific studies about impurities of these heavy metals in pharmaceutical products are treated very marginally. It seems that nowadays lead and cadmium impurities are not a popular problem in scientific literature; however, herbal plants in traditional herbal medicinal products (THMPs) can especially be a potential source of these elements impurities [1]. One of the most well-known and popular THMPs among the European population is extract derived from *Althaea officinalis* L., radix (marshmallow root). This herbal plant has been used since ancient times in different European countries [2]. The THMPs with this ingredient are still widely applied phytopharmaceuticals for the treatment of pharyngeal or oral and associated dry cough [2]. The marshmallow root consists of (un)peeled dried root of *Althaea officinalis* L. [3]. The procedures for the preparation of marshmallow syrup are usually similar, for example, based on Polish Farmakopea (2002) [4]: “*Sirupus althaeae* is prepared by maceration of 5 parts of roots with 1 part of ethanol (760 g/L) and 40 parts of purified water for 3 h without stirring. In macerate obtained 64 parts of sucrose and 0.1 part of benzoic acid are solved”. This kind of pharmaceutical product is widely applied for irritation of the pharyngeal and oral mucosa and associated dry cough [5], throat irritation [6,7], and against cough [8]. Even though this product is extremely popular, there is a lack of studies about elemental impurities (EI) in this kind of product. Only one article describes the levels of Pb (2.93 mg/kg) and Cd (0.26 mg/kg), but in leaves of *A. officinalis* L. [9], not in the final THMP.

It should be mentioned that exposure to Pb and Cd may cause serious health effects—especially [10,11,12] neurological, immunological, cardiovascular, reproductive, developmental, and also renal issues. There is no doubt that this toxic metal is very harmful for people [12]. The problem with the persistence of this element exposure indicates the need to identify sustainable solutions to define lead exposures [13]. Lead can also be present as environmental impurities in plants because it may be taken up by plant roots [14]. Additionally, because this element is taken up by plants, the toxicity level (in the range of 0.5–1 mg/kg dry plant material) can be a serious problem for health [15]. On the other hand, a second important heavy metal, cadmium, can impair skeletal and reproductive systems, and cause kidney damage and other problems. Cd is readily taken up by plants, hence the potential problem is that the toxicity level (0.5–1 mg/kg dry plant material), whereas crop plants tolerate at least tenfold of that concentration in tissue [15].

Hence, comprehensive health assessment of Pb and Cd impurities in THMPs with *A. officinalis* L. is required. For this purpose, the best strategy is based on the ICH Q3D (International Council for Harmonization of Technical Requirements for Pharmaceuticals for Human Use) guideline about acceptable limits of EI [1]. In this guideline, Pb, Cd, and other heavy metals (As and Hg) are classified as a first class because these elements are human toxicants that have limited or no application in the pharmaceutical industry [1]. 

Hence, our work aimed at a comprehensive assessment of Pb and Cd impurities (based on the ICH Q3D guideline mentioned earlier) in THMPs with *Althaea officinalis* L., radix, available in Polish pharmacies. Our studies included all available THMPs in the market with marshmallow root extracts in Poland (*n* = 10). The justification for the choice of this study was the lack of appropriate investigations about Pb and Cd impurities in such pharmaceutical products. Additionally, there are common opinions among the Polish population about the alleged PTE impurities in THMPs available in Poland, without any scientific background.

## 2. Materials and Methods

### 2.1. Chemicals

For the experimental work, demineralized water was applied during sample processing. Nitric acid (65%) was of spectroscopic grade (Merck SupraPur, Darmstadt, Germany). Standard solution for Pb was standard solution traceable to SRM from NIST—Pb(NO_3_)_2_ in 0.5 mol/L HNO_3_ and 1000 mg/L Pb CertiPUR^®^ (catalog product: 1.19777.0500) and cadmium (Cd standard solution traceable to SRM from NIST–Cd(NO_3_)_2_ in 0.5 mol/L HNO_3_, 1 g/L Cd CertiPUR^®^, catalog product: 1.19777.0500) were prepared by dilution of certified standard solutions, 1 mg/L MERC of corresponding metal ions. The purchased certified reference material (CRM; BCR-482; IRMM, Belgium) was material prepared from lichen. Additionally, the second certified reference material (Corn Flour, INCT-CF-3) was purchased from the Institute of Nuclear Chemistry and Technology—Department of Analytical Chemistry (Warsaw, Poland).

### 2.2. Samples

THMPs containing *Althaea officinalis* L., radix were collected from pharmacies in Poland. In our studies, we analyzed all available THMPs in Poland with marshmallow root extracts (*n* = 10). All samples were denoted by letters (A–J). It should be noted that only a few independent manufacturers produce these kinds of pharmaceutical products in Poland. The investigated products were over-the-counter medicines collected from pharmacies in Kraków, Niepołomice, Bochnia, Wieliczka, and Rzeszów in 2021 (April–May). All investigated THMPs were syrups and contained *A. officinalis* L. (root). The preparation in all cases was the same. The detailed information about each product (based on manufacturer information) is described in more detail in Table 1.

### 2.3. Determination of Pb and Cd

**Figure 1:**
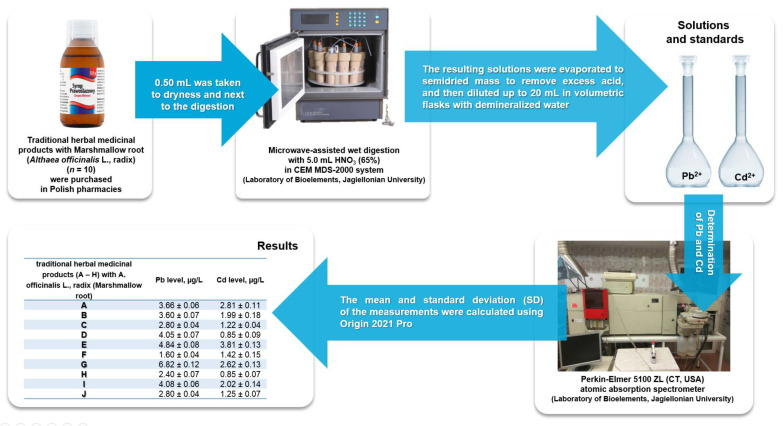
The workflow of the applied toxicological assessment of lead and cadmium in investigated traditional herbal medicinal products with *Althaea officinalis* L., radix (marshmallow root) collected from pharmacies in Poland.

The acid digestion of samples was applied using a microwave oven MDS 2000 (CEM, American Laboratory Trading, East Lyme, CT, USA). Each THMP with *Althaea officinalis* L., radix was homogenized. From each sample, 0.5 mL was measured (0.5 mL samples containing 20% ethyl alcohol were taken to dryness before digestion), poured into Teflon vessels, and digested (1 h) with 5.0 mL of concentrated nitric acid (HNO_3_, 63%). After this operation, all samples were cooled to a room temperature (25 °C). The final volume was 20 mL. Five replications were performed for all samples to increase the precision of the results. The time–temperature program in the graphite furnace atomic absorption spectrometer for Pb and Cd determination is presented in Table 2. The applied instrumental conditions for Pb and Cd determination by ET AAS was presented in Table 3. The workflow of each step is presented in Figure 1.

The analytical calibration function includes levels 0.0; 1.0; 2.0; 5.0; 10.0 µg/L for Pb, and 0.0; 0.5; 1.0; 2.0 µg/L for Cd. The values of correlation coefficients (R) were 0.998 for Pb and 0.998 for Cd, which indicated that the analyses were both precise and accurate.

The obtained values for recoveries were 98.2% and 97.5% for Pb and Cd, respectively.

The calculated limits of detection (LODs) were 0.46 µg/L for Pb and 0.16 µg/L for Cd. The calculated limits of quantification (LOQs) were 0.96 µg/L for Pb and 0.35 µg/L for Cd.

The CRM was prepared from lichen (BCR-482; IRMM, Geel, Belgium) and corn flour, INCT-CF-3. Until the analysis, samples of CRM were dried at 70 °C for 24 h. After drying, the samples were transferred for microwave digestion (digestion was carried out using a programmable microwave oven (model MDS-2000; CEM Corp., Matthews, NC, USA). In the first step, 5 mL of nitric acid (concentration 65%) was added to 300 mg of the sample in Teflon reaction vessels and left to predigest for 24 h. In the next step, digestion was carried out, and after cooling the vessels, the extracts were transferred to Sarstedt vessels and completed with demineralized water to a total volume of 15 mL. Samples prepared were analyzed using a Perkin-Elmer 5100 ZL atomic absorption spectrometer with a graphite furnace. The declared value of Pb was 40.9 ± 1.4 mg/kg and the obtained value was 38.05 ± 0.23 mg/kg for BCR-482; IRMM. The declared value of Pb was 0.052 ± 0.009 mg/kg and the obtained value was 0.050 ± 0.009 mg/kg for corn flour, INCT-CF-3. The analysis of the mentioned certified reference materials was helpful for assessing the traceability of the results. The applied methodology was similar to our previous studies using the same apparatus [10,16,17,18,19,20,21].

### 2.4. Data Analysis

Obtained results were calculated using the scientific statistical software Excel 2010 (Microsoft Office) and Origin 2021 Pro.

### 2.5. Toxicological Assessment of Heavy Metals Impurities in Investigated Pharmaceutical Products

**Figure 2:**
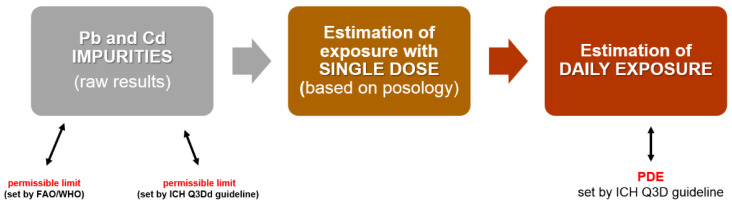
The block diagram of applied strategy for toxicological assessment of Pb and Cd impurities in THMPs with *Althaea officinalis* L., radix (marshmallow root) available in Poland.

The strategy for our toxicological assessment of investigated PTE impurities is shown schematically in Figure 2.

The first step was the preparation of raw results impurities for each THMP. The results were compared with the permissible limit set by FAO/WHO for medicinal herbs in different countries. The next step was the assessment of exposure of lead impurities after the application of THMP. For this purpose, the first step was the estimation of Pb levels in a single administration (ng/single dose) based on posology. The last step was to estimate the daily dose of PTE (ng/day) and compare with permitted daily exposure (PDE) based on the ICH Q3D guideline.

## 3. Results

### 3.1. The Lead and Cadmium Impurities (Raw Results) in Traditional Herbal Medicinal Products with A. officinalis L., radix (Marshmallow Root) in Polish Pharmacies

The results of all analyzed samples of THMP samples (A–H) are presented in Table 4.

### 3.2. The Assessment of Exposure of Pb and Cd Impurities after Application of THMPs with A. officinalis L., radix (Marshmallow Root) Available in Polish Pharmacies

The comprehensive toxicological assessment of investigated PTE impurities also requires estimation of exposure after oral application. Hence, based on the posology for each product (compare with Table 1), the estimated exposure after a single administration (ng/single dose) and estimated maximum daily exposure of Pb and Cd impurities (ng/day) are presented in Table 5.

## 4. Discussion

The general characteristics indicate that lead impurities were present at a relatively low level (mean = 3.73 μg/L) in all analyzed samples (1.62–6.74 μg/L). The lowest level of Pb was in sample F (1.62 ± 0.13 μg/L), and the highest level was observed in sample G (6.74 ± 0.24 μg/L). It should be noted that there are limited studies about Pb level in *A. officinalis* L., radix (marshmallow root). Azizov et al. [22] and Esmail et al. [23] described that the level of Pb was 26.1 mg/kg in roots of *A. officinalis* L., radix collected in Syrdarinsk District of Uzbekistan. As was mentioned in the introduction, lead may be taken up by plant roots [14]. Additionally, because it is readily taken up by plants, an important problem can be that the toxicity level is in the range of 0.5–1 mg/kg dry plant material, whereas crop plants tolerate at least tenfold of that concentration in tissue [15]. In mentioned studies, the level of Pb was 26.1 mg/kg in roots, which indicates that safe levels have been seriously exceeded (>1 mg/kg dry plant material). However, because the analyzed samples were syrups, the above-mentioned results (the content in the roots) cannot be directly compared with our results. Nevertheless, the cited data justify the need for this type of research, as this plant can accumulate lead at quite a significant level. From a regulatory point of view, in all investigated samples, the Pb levels were below the permissible limit set by FAO/WHO for medicinal herbs and plants in different countries (10^4^ µg/kg [24]). Additionally, considering the concentration limits for Pb as impurities in pharmaceuticals via oral route recommended by the ICH Q3D guideline (0.5 μg/g [1]), all of the THMPs meet the guidelines.

Similar results were observed for cadmium; the Cd impurities were present at a very low level (mean = 1.88 μg/L) in all investigated samples (0.85–3.81μg/L). The highest Cd level was observed in sample E (3.81 ± 0.13 μg/L), and the lowest level was in samples D (0.85 ± 0.09 μg/L) and H (0.85 ± 0.07 μg/L). As in the case of Pb, there are limited studies about Cd levels in *A. officinalis* L., radix (marshmallow root). Azizov et al. [22] and Esmail et al. [23] described that the level of Cd was 0.83 mg/kg in roots of *A. officinalis* L., radix collected in Syrdarinsk District of Uzbekistan. This element is readily taken up by plants, and an important problem can be that the toxicity level is in the range of 0.5–1 mg/kg dry plant material, whereas crop plants tolerate at least ten-fold of that concentration in tissue [15]. The above-mentioned results cannot be compared with our obtained results because we analyzed final pharmaceutical products (diluted products, not plants). From a regulatory point of view, in all investigated samples, the Cd levels were below the permissible limit set by FAO/WHO for medicinal herbs and plants in different countries (300 µg/kg [22]). Additionally, considering the ICH Q3D guidelines for final drugs (Cd limits: 0.5 μg/g [1]), the standards are also met. However, more comprehensive toxicological assessment (daily exposure) is needed in this situation (the present heavy metals require additional evaluation [1]).

The obtained results (Table 5) of daily exposure of lead (60.95–304.29 mg 10^−6^/day) are variable. Based on the Integrated Exposure Uptake Biokinetic (IEUBK) Model [25], PDE value was established as 5.0 μg/day [1]. Hence, it can be concluded that all investigated products are characterized by results below PDE value (highest result: 0.304 mg 10^−6^/day—sample G). Additionally, the estimated daily exposure levels for Cd are variable (21.25–228.6 mg 10^−6^/day). The main point for toxicological assessment of Cd by oral route is renal toxicity [26]; hence the renal toxicity was applied by EMA to define the oral PDE value (5.0 μg/day [1]). This was justified by the fact that many studies about oral exposure to cadmium in rats and mice showed no evidence of carcinogenicity. Our results indicate that all analyzed samples are characterized by results extremely lower than PDE value for cadmium.

## 5. Conclusions

Our studies are extremely important for regulatory purposes (regulatory toxicology), especially for the pharmaceutical industry. Conducted studies show that lead (1.62–6.74 μg/L) and cadmium (0.80–3.81 μg/L) impurities as raw results were at a very low level in all analyzed THMPs with *Althaea officinalis* L., radix. Additionally, all results were below the permissible limit set by FAO/WHO for medicinal herbs and plants in different countries (10,000 µg/kg for Pb and 300 µg/kg for Cd). The contents of these elements in a single dose were also at a very low level; hence, there is no potential hazard for people. Additionally, the estimated daily intake of Pb and Cd impurities compared to the PDE value confirm all samples safety. Hence, it can be summarized that all samples meet the standards of the ICH Q3D guideline due to the PTE impurities.

The broader toxicological assessment considering other heavy metals (Hg and As) in THMPs available in European pharmacies will be valuable in future studies. It should be noted that we analyzed all available products in Poland (*n* = 10) but this could be a limit for global interpretation (in the future, other researchers should also include products from outside Poland, which was impossible for us at this time).

## Figures and Tables

**Table 1:** The descriptions of the investigated THMPs with marshmallow root collected from pharmacies in Poland.

Descriptor	Sample									
	A	B	C	D	E	F	G	H	I	J
Amount of API in 10 mL of syrup	0.33	0.27	0.29	0.21	0.28	0.31	0.30	0.25	0.21	0.29
Posology	Orally: 15 mL, 3 times daily	Orally: 10–15 mL, 3 times daily	Orally: 15 mL, 3 times daily	Orally: 15 mL, 3 times daily	Orally: 15 mL, 3–4 times daily	Orally: 15 mL, 3–4 times daily	Orally: 15 mL, 3 times daily	Orally: 5 mL, 5 times daily	Orally: 15 mL, 3 times daily	Orally: 10–15 mL, 3 times daily
Density, g/mL	1.29	1.31	1.26	1.28	1.32	1.30	1.295	1.33	1.28	1.26
Lot number	20920	02AF0620	01AF0820	01AF0920	21220	61020	10420	454201	01AF0920	01AF0820
License	IL-0692/LN	12180	Not applicable	Not applicable	IL-5954/LN	IL-4746/LN	8147	Not applicable	Not applicable	Not applicable
Classification	OTC	OTC	Diet supplement	Diet supplement	OTC	OTC	OTC	Medical product	Diet supplement	Diet supplement

**Table 2:** Time–temperature program in the graphite furnace atomic absorption spectrometer for Pb and Cd determination.

**Pb**	**Step**	**Temperature [°C]**	**Ramp [s]**	**Hold [s]**	**Gas Flow [mL/min]**
	1	120	1	30	250
	2	950	10	20	250
	3	1450	0	5	0
	5	2400	1	2	250
**Cd**	**Step**	**Temperature [°C]**	**Ramp [s]**	**Hold [s]**	**Gas Flow [mL/min]**
	1	120	10	25	250
	2	300	5	15	250
	3	1600	0	3	0
	5	2400	1	2	250

**Table 3:** The applied instrumental conditions for Pb and Cd determination by ET AAS.

Operating Parameters	Pb	Cd
Wavelength, nm	283.3	228.8
Lamp current, mA	8	5
Slit width, nm	0.7	0.7
Optimum working range, µg/kg	1.0–10.0	0.02–0.20

**Table 4:** The results of Pb and Cd impurities in analyzed THMPs (A–H) with *A. officinalis* L., radix (marshmallow root) from Polish pharmacies as raw results.

Traditional Herbal Medicinal Products (A–H) with *A. officinalis* L., radix (Marshmallow Root)	Pb Level, µg/L	Cd Level, µg/L
A	3.66 ± 0.06	2.81 ± 0.11
B	3.60 ± 0.07	1.99 ± 0.18
C	2.80 ± 0.04	1.22 ± 0.04
D	4.05 ± 0.07	0.85 ± 0.09
E	4.84 ± 0.08	3.81 ± 0.13
F	1.60 ± 0.04	1.42 ± 0.15
G	6.82 ± 0.12	2.62 ± 0.13
H	2.40 ± 0.07	0.85 ± 0.07
I	4.08 ± 0.06	2.02 ± 0.14
J	2.80 ± 0.04	1.25 ± 0.07

**Table 5:** The calculated exposure of Pb and Cd in single administration and maximal daily dose for each THMP with *A. officinalis* L., radix (marshmallow root) available in Polish pharmacies.

Sample	Estimated Oral Exposure of Pb Based on Posology	
	Single Administration, mg 10^−6^/single Dose	Maximum Daily Dose, mg 10^−6^/day
A	55.874	167.71
B	53.514	160.53
C	41.223	123.66
D	61.844	185.52
E	70.144	280.56
F	25.113	100.44
G	101.43	304.29
H	12.186	60.948
I	61.844	185.52
J	41.223	123.66
**Sample**	**Estimated Oral Exposure of Cd Based on Posology**	
	**Single Administration, mg 10^−6^/single Dose**	**Maximum Daily Dose, mg 10^−6^/day**
A	42.150	126.45
B	29.850	89.55
C	18.299	54.90
D	12.750	38.25
E	57.151	228.60
F	21.300	85.20
G	39.310	117.90
H	4.251	21.25
I	10.101	50.50
J	6.250	31.25

## Data Availability

Not applicable.
